# TAP Hunter: a SVM-based system for predicting TAP ligands using local description of amino acid sequence

**DOI:** 10.1186/1745-7580-6-S1-S6

**Published:** 2010-09-27

**Authors:** Tze Hau Lam, Hiroshi Mamitsuka, Ee Chee Ren, Joo Chuan Tong

**Affiliations:** 1 Laboratory of Immunogenetics and Viral Host-Pathogen Genomics, Singapore Immunology Network, 8A Biomedical Grove, #03-06, Immunos, Singapore 138648; 2 Bioinformatics Center, Institute for Chemical Research, Kyoto University, Gokasho, Uji 611-0011, Japan; 3 Department of Microbiology, Yong Loo Lin School of Medicine, National University of Singapore, 8 Medical Drive, Singapore 117597; 4 Department of Biochemistry, Yong Loo Lin School of Medicine, National University of Singapore, 8 Medical Drive, Singapore 117597; 5 Data Mining Department, Institute for Infocomm Research, 1 Fusionopolis Way, #21-01 Connexis, South Tower, Singapore 138632

## Abstract

**Background:**

Selective peptide transport by the transporter associated with antigen processing (TAP) represents one of the main candidate mechanisms that may regulate the presentation of antigenic peptides to HLA class I molecules. Because TAP-binding preferences may significant impact T-cell epitope selection, there is great interest in applying computational techniques to systematically discover these elements.

**Results:**

We describe TAP Hunter, a web-based computational system for predicting TAP-binding peptides. A novel encoding scheme, based on representations of TAP peptide fragments and composition effects, allows the identification of variable-length TAP ligands using SVM as the prediction engine. The system was rigorously trained and tested using 613 experimentally verified peptide sequences. The results showed that the system has good predictive ability with area under the receiver operating characteristics curve (A_ROC_) ≥0.88. In addition, TAP Hunter is compared against several existing public available TAP predictors and has showed either superior or comparable performance.

**Conclusions:**

TAP Hunter provides a reliable platform for predicting variable length peptides binding onto the TAP transporter. To facilitate the usage of TAP Hunter to the scientific community, a simple, flexible and user-friendly web-server is developed and freely available at http://datam.i2r.a-star.edu.sg/taphunter/.

## Background

The binding of peptides to human leukocyte antigen (HLA) class I molecules is a prerequisite for CD8+ T cell response. Majority of these peptides are generated in the cytosol by proteosomal cleavage of endogenous proteins [1]. The degraded peptides, preferably 9-18 amino acids in length, are transported into the lumen of the endoplasmic reticulum (ER) by the transporter associated with antigen processing (TAP) for loading on HLA class I molecules [2,3]. The ligated HLA class I complexes then leave the ER and are transported to the cell surface for presentation to T cell receptors [4]. Defects in TAP genes can severely impair peptide transport into the ER, and result in reduced surface expression of HLA class I molecules [5].

The substrate specificity of TAP has been examined in several studies. It is now known that hydrophobic aromatic residues are preferred at the C-terminus, positions (p) 3, and p7; hydrophobic or positively charged residues are preferred at p2; aromatic or acidic residues are preferred at p1; and proline is disfavored at p1 and p2 [5-7]. Different HLA class I alleles exhibit different TAP-dependencies. HLA-A2 is reportedly the least TAP-dependent; B7 can bind to other mechanisms besides TAP transport; while A3 is predominantly TAP dependent [8]. As such, improved understanding of TAP selectivity is important for elucidating its role in regulating the supply of peptides to HLA class I molecules. This is also crucial for the design of T cell-based vaccines for infectious diseases, autoimmune disorders, transplantation and cancer.

To date, a variety of computational methods have been developed to predict TAP-binding peptides. Daniel and coworkers [9] applied artificial neural networks (ANN) to simulate TAP binding experiments. Zhang *et al. *[10] combined ANN and hidden Markov models to predict peptide binding to human TAP. Doytchinova and colleagues [11] developed an additive QSAR model for peptides binding to TAP molecule. Bhasin and Raghava [12] utilized a cascade support vector machines (SVM)-based method to predict the binding affinities of TAP ligands, while Peters *et al*. [13] and Diez-Rivero *et al. *[14] reported the use of stabilized matrix method and SVM-based system, respectively, to predict both nonamer and variable length TAP ligands. Although numerous studies have shown the importance of sequence locality in TAP transport [12], none of the existing systems have exploited localized amino acid effect for predicting TAP binding affinity of peptides.

Here we report TAP Hunter, a web-based computational system for predicting TAP ligands using SVM as the discrimination engine. A novel data encoding scheme, based on sequence locality and composition effects, allows the system to model essential features in peptides that can bind to the TAP translocator. This simple method allows us to predict TAP ligands with an accuracy that is better than existing approaches based on full-length sequences.

## Methods

### Data

The dataset consists of 896 peptide sequences. In this list, to use the same dataset as those of the existing work [12,13], we first focused on 276 TAP binding and 94 non-binding nonamer peptides, which were derived from TAP binding assays [10]. We used them for 5-fold cross validation (CV) to select the best model out of the 48 models that we examined on different amino acid positions (see Table 1 for selected models). We then trained the optimized model using all 276 binders and 94 non-binders once again, and its performance was assessed using three independent datasets: i) 91 TAP binding and 32 non-binding nonamer peptides derived from TAP binding assays [9]; and ii) 38 recently elucidated nonamer peptides from TAP dependent HLA-A1, A3, A11, A24 and B27 [15], and 12 nonamer peptides from TAP-deficient LCL721.174 cell line [16].

**Table 1:** Performance evaluation of SVM models using different peptide localities (selected outputs are shown)

No of a.a.	Model No.	a.a. positions used in modeling	ACC		AROC	
			
			5-fold CV	Independent testing	5-fold CV	Independent testing
	1	2, 3	0.80	0.76	0.80	0.75
2	2	2, 9	0.76	0.77	0.80	0.83
	3	3, 9	0.79	0.78	0.81	0.86

	4	1, 2, 3	0.79	0.77	0.83	0.78
3	5	1, 2, 9	0.82	0.82	0.88	0.86
	6	1, 3, 9	0.80	0.77	0.84	0.85
	7	2, 3, 9	0.82	0.83	0.87	0.88

	8	1, 2, 3, 7	0.79	0.73	0.84	0.76
	9	1, 2, 3, 8	0.79	0.79	0.82	0.77
	10	1, 2, 3, 9	0.84	0.82	0.88	0.88
	11	1, 2, 7, 9	0.81	0.75	0.87	0.83
4	12	1, 2, 8, 9	0.80	0.78	0.86	0.86
	13	1, 3, 7, 9	0.81	0.79	0.85	0.86
	14	1, 3, 8, 9	0.82	0.81	0.83	0.86
	15	2, 3, 7, 9	0.83	0.76	0.89	0.83
	16	2, 3, 8, 9	0.78	0.82	0.85	0.88

	17	1, 2, 3, 7, 8	0.79	0.76	0.84	0.74
	18	1, 2, 3, 8, 9	0.81	0.82	0.86	0.89
5	19	1, 2, 7, 8, 9	0.82	0.76	0.87	0.83
	20	1, 2, 3, 7, 9	0.82	0.77	0.88	0.86
	21	1, 3, 7, 8, 9	0.80	0.80	0.85	0.83
	22	2, 3, 7, 8, 9	0.82	0.79	0.86	0.86

	23	1, 2, 3, 4, 5, 6	0.76	0.8	0.83	0.80
6	24	1, 2, 3, 7, 8, 9	0.82	0.79	0.85	0.86
	25	4, 5, 6, 7, 8, 9	0.78	0.69	0.80	0.59

	26	1, 2, 3, 4, 7, 8, 9	0.80	0.75	0.85	0.84
7	27	1, 2, 3, 5, 7, 8, 9	0.81	0.76	0.85	0.86
	28	1, 2, 3, 6, 7, 8, 9	0.81	0.77	0.86	0.84

	29	1, 2, 3, 5, 6, 7, 8, 9	0.81	0.82	0.86	0.85
8	30	1, 2, 3, 4, 6, 7, 8, 9	0.80	0.80	0.85	0.84
	31	1, 2, 3, 4, 5, 7, 8, 9	0.78	0.79	0.83	0.85

9	32	1, 2, 3, 4, 5, 6, 7, 8, 9	0.79	0.78	0.84	0.83

### Support vector machines

SVMs are a type of supervised statistical machine-learning techniques based on the structural risk minimization principle used for classification and regression. In this work, SVM is used to binary classify the peptides into TAP- binding or TAP non-binding. Suppose S = {(*x_1_, y_1_*) … (*x_i_, y_i_*)} is a set of *i* training samples, where *x* is the feature vectors in d-dimensional domain      (*x_i_ ∊ R^d^*) representing an individual peptide and *y_i_ ∊ {1,-1}*. For a binary classification, the kernel function is utilized to map the input feature vectors into a higher dimensional feature space. Within this feature space, SVM modelling will locate an optimal hyperplane separating the vectors into two distinct categories. The decision function for the classifier can be written as
				

*α_i_* is solved by quadratic programming subjected to 0≤ *α_i_* ≤*C* condition,  where *C* is the parameter to control the trade-off between the margin and training error. *K* represents the kernel function while *sgn* is the sign of the argument in the form of -1 or 1. If the function of a test instance is greater than zero, it will be tagged as positive case while a function value of less than zero is presented as negative case. This concept of kernel function mapping allows SVM to model very complex precincts and thus enable SVMs to easily handle non-linear data. Though there are many different type kernels proposed by researchers, the commonly used and broadly relevance to many applications are the linear, polynomial, radial basis functions and sigmoid kernel functions.

### Model building and evaluation

TAP Hunter was implemented using the SVM-Light package [17]. The system employs the Radial Basis Function (RBF) kernel for SVM training. We also explored linear and polynomial kernel functions but they did not achieve higher performance levels (data not shown). The inputs to the SVM are binary strings or feature vectors representing encoded representations of physicochemical properties previously reported as significant for TAP binding [12]. These include hydrophobicity, aromaticity, charges and residue weight. It has been reported that the N- and C-terminal residues of TAP ligands contribute to most of the binding interactions [12]. Using the above features, truncation analysis was performed to examine the contribution of each and every peptide position to binding. 5-fold cross-validation (CV) was performed to assess the stability of the derived models. Finally, the performance of each models were assessed using sensitivity (SE), specificity (SP), accuracy (ACC) and the area under the Receiver Operating Characteristic curve (AROC) as previously described [18].

## Results

### System performance

The robustness of TAP Hunter using different sequence localities as inputs for training has been estimated for 5-fold CV (Table 1). The best model was achieved using descriptors derived from peptide positions N+1, N+2, N+3 and C (model 10; ACC=0.84 and AROC=0.82 for 5-fold CV; ACC=0.88 and AROC= 0.88 for Testing dataset i), consistent with existing studies that these amino acid positions are crucial for binding [12]. 

### Comparison with existing methods

**Figure 1:**
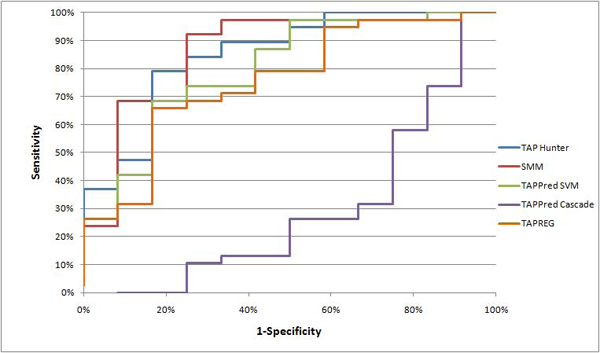
Mean AROC curve evaluation for various TAP predictors

We benchmarked the performance of TAP Hunter against four existing techniques: TAPPred (SVM) [12], TAPPred (Cascade SVM) [12], Stabilized matrix method (SMM) [13] and TAPREG [14] using an independent dataset of 50 recently elucidated nonamer peptides (Testing dataset ii). Among them, only SMM and TAPREG have the capacity to predict arbitrary length ligands. Each of these techniques has its own defined threshold for discriminating TAP-binding ligands. For objective evaluation of the systems’ performance, the threshold independent AROC was adopted in this study. And to illustrate the observed AROC difference between TAP Hunter and each of the current methods is statistically significant; we used bootstrapping to randomly sample the testing dataset to into smaller sizes for statistical inference. As shown in Figure 1, the sequence locality approach as implemented in TAP Hunter consistently outperforms or is comparable to all existing techniques evaluated in this study – TAP Hunter: mean AROC=0.85 (± 0.018 95% CI); Stabilized matrix method (SMM): mean AROC=0.86 (± 0.023 95% CI); TAPPred (SVM): mean AROC=0.80 (± 0.023 95% CI); TAPPred (Cascade SVM): mean AROC=0.28 (± 0.022 95% CI); TAPREG: mean AROC=0.76 (± 0.029 95% CI). The computed p-values on the observed AROC difference between TAP Hunter and the respective methods are shown in Table 2. The results indicate that, overall, TAP Hunter is capable of screening peptides that could be transported by TAP using local description of amino acid sequence. There are also algorithms that integrate different sub-components of the antigen processing and presentation pathway such as proteasome, TAP, and HLA [19,20]. However, we did not benchmark these systems as only the aggregate scores of prediction are provided.

**Table 2:** p-values for the observed AROC difference between TAP Hunter and each of the existing TAP predictors for nonamers ligands predictions

	TAPPred SVM	TAPPred Cascade	SMM	TAPREG
TAP Hunter	8.2x10^-4^	2.2x10^-16^	Not Significant	5.1x10^-8^

### Web-server implementation and description

The execution of the TAP Hunter web-server comprises of two segments, the front and the back end. The front end, written in HTML and JavaScript, consists of the web-interface designed for user input sequence(s) as well as the references and databases used for the collection of the training and evaluation datasets. The back end administration is run by several modules (written in Perl, JavaScript, HTML, CGI and Java) for (i) the input sequence(s) error assessment, (ii) the cleavage of protein sequence into the user defined peptide length, (iii) the generation sequence feature vectors, the operation of SVM-light package and (iv) output of results. TAP Hunter has been rigorously tested on Internet Explorer (IE) and Mozilla Firefox browsers and is expected to perform on other major web browsers. Typically the processing time required to perform TAP-peptide binding affinity prediction operation for 566 nonamer peptides is less than 30 seconds. 

**Figure 2:**
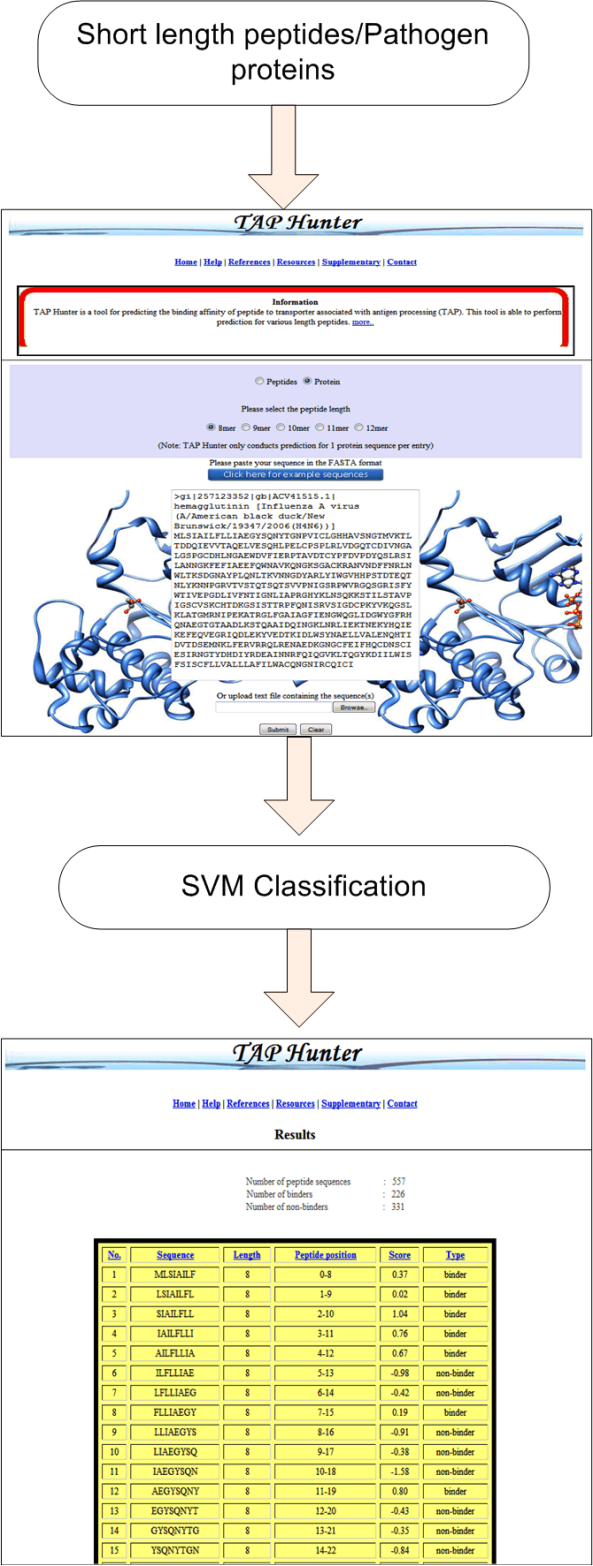
**Snapshot of the prediction process using TAP-Hunter** The results are presented as a sortable table according to the selected parameters at the top.

The operation of TAP Hunter is simple, flexible and user-friendly (Figure 2). TAP Hunter allows prediction for both short-length peptides and pathogen proteins to be screened for TAP binding peptides. Users either input sequence(s) in fasta format in the textbox or upload text file containing the sequence(s) to perform prediction. For short length peptide prediction, the maximum peptide length allowed is 21 amino acid residues while for protein sequence type prediction is limited to a maximum peptide length of 12 amino acid residues.

## Discussion and conclusion

The complex molecular mechanism involved in antigen processing and presentation pathway has impeded our capability to predict the adaptive nature of immune responses confidently. Discovery through experimental evaluation is expensive and time-consuming. Yet, usage of computational methods to complement laboratory experiments is likely to expedite the knowledge discovery in immunology. Particularly in recent years, we have seen increased attempts to simulate the cell-mediated immune system by integrating the proteasome, TAP, and HLA components of the antigen processing and presentation pathway [19-22]. A study by Doytchinova and colleagues in 2004 has shown that TAP pre-selection could reduce the number of non-binders from 10% (TAP-independent) to 33% (TAP-dependent). In this aspect, TAP Hunter derives its feature vectors from the N- and C- terminal positions of TAP ligands that are known to exhibit binding motifs and most heavily influence the TAP binding affinity [5-7]. Our investigation has shown that this innovative solution is equally adept or even superior in discriminating nonamer TAP binding peptides than all current nonamer TAP predictors. Further refinement in the feature selection procedure may enable the development of TAP Hunter into a practical tool for pre-selecting T cell epitopes. 

## Competing Interests

The authors declare no competing financial interests.

## Authors’ Contributions

JCT conceived the study. THL designed and performed the experiments. JCT, THL, HM and ECR analyzed the data and wrote the paper. 
